# Building a bigger tent in point-of-care ultrasound education: a mixed-methods evaluation of interprofessional, near-peer teaching of internal medicine residents by sonography students

**DOI:** 10.1186/s12909-018-1437-2

**Published:** 2018-12-27

**Authors:** Christopher J. Smith, Tabatha Matthias, Elizabeth Beam, Kathryn Wampler, Lea Pounds, Devin Nickol, Kristy Carlson, Kimberly Michael

**Affiliations:** 1 Department of Internal Medicine, University of Nebraska Medical Center, Section of Hospital Medicine, 986430 Nebraska Medical Center, Omaha, NE 68198-6430 USA; 2 University of Nebraska Medical Center, Interprofessional Academy of Educators, 987115 Nebraska Medical Center, Omaha, NE 68198-7115 USA; 3 Department of Medical Imaging & Therapeutic Sciences, Diagnostic Medical Sonography Program, University of Nebraska Medical Center, 984545 Nebraska Medical Center, Omaha, NE 68198-4545 USA; 4 Department of Health Promotion, University of Nebraska Medical Center, 984395 Nebraska Medical Center, Omaha, NE 68198-4320 USA; 5 Department of Internal Medicine, Division of General Internal Medicine, University of Nebraska Medical Center, 986430 Nebraska Medical Center, Omaha, NE 68198-6430 USA; 6 Department of Internal Medicine, Section of Hospital Medicine, 986430 Nebraska Medical Center, Omaha, NE 68198-6430 USA

**Keywords:** Point-of-care ultrasound, Peer-teaching, Interprofessional education

## Abstract

**Background:**

Point-of-care-ultrasound (POCUS) training is expanding in undergraduate and graduate medical education, but lack of trained faculty is a major barrier. Two strategies that may help mitigate this obstacle are interprofessional education (IPE) and near-peer teaching. The objective of this study was to evaluate a POCUS course in which diagnostic medical sonography (DMS) students served as near-peer teachers for internal medicine residents (IMR) learning to perform abdominal sonography.

**Methods:**

Prior to the IPE workshop, DMS students participated in a train-the-trainer session to practice teaching and communication skills via case-based simulation. DMS students then coached first-year IMR to perform POCUS examinations of the kidney, bladder, and gallbladder on live models. A mixed-methods evaluation of the interprofessional workshop included an objective structured clinical exam (OSCE), course evaluation, and qualitative analysis of focus group interviews.

**Results:**

Twenty-four of 24 (100%) IMR completed the OSCE, averaging 97.7/107 points (91.3%) (SD 5.2). Course evaluations from IMR and DMS students were globally positive. Twenty three of 24 residents (96%) and 6/6 DMS students (100%) participated in focus group interviews. Qualitative analysis identified themes related to the learning environment, scanning technique, and suggestions for improvement. IMR felt the interprofessional training fostered a positive learning environment and that the experience complimented traditional faculty-led workshops. Both groups noted the importance of establishing mutual understanding of expectations and suggested future workshops have more dedicated time for DMS student demonstration of scanning technique.

**Conclusion:**

An interprofessional, near-peer workshop was an effective strategy for teaching POCUS to IMR. This approach may allow broader adoption of POCUS in medical education, especially when faculty expertise is limited.

**Electronic supplementary material:**

The online version of this article (10.1186/s12909-018-1437-2) contains supplementary material, which is available to authorized users.

## Background

Point-of-care ultrasound (POCUS) is the application of portable ultrasound technology by clinicians to aid in real-time diagnosis, management, and treatment decisions. POCUS is a powerful adjunct to the physical exam with a wide variety of clinical applications [1]. In recent years, POCUS education has grown rapidly in undergraduate [2, 3] and graduate medical education. Inspired by emergency medicine [4] and critical care medicine [5], internal medicine residency programs have become fertile grounds for POCUS education. A study in 2013 found that 25% of internal medicine residency programs in the United States had formal POCUS curriculum, with another quarter planning to implement a curriculum in the next year [6]. A survey of Canadian program directors demonstrated similar findings [7]. Despite this growth, there remains significant training gaps in POCUS skills among internal medicine faculty and residents [7, 8]. Several national studies of educational leaders have identified lack of adequately trained faculty as a major barrier to POCUS curriculum expansion [6, 7, 9].

Two pedagogical approaches that may mitigate lack of POCUS-trained faculty include peer teaching and interprofessional education. Peer teaching occurs when people from similar social groups work cooperatively to learn from one another [10]. The term “near-peer teaching” is sometimes used when those involved have different levels of training or seniority [11]. Peer-led teaching has several potential advantages, including creation of a more comfortable learning environment; enhancement of leadership, communication, and organizational skills; and curricular sustainability when teaching resources are limited [12]. Peer teaching of sonography skills has shown promise among medical [13] and under-graduate students [14], but it is unclear if this can translate to residency programs which have unique challenges, including competing curricular priorities, scheduling conflicts, and a lack of trained house officers to serve as peer-mentors.

Interprofessional education (IPE) is defined as education that “occurs when students from two or more professions learn about, from, and with each other to enable effective collaboration and improve health outcomes.” [15] IPE is supported by a wide-spectrum of professional organizations via the Interprofessional Education Collaborative (IPEC) who has published core competencies for interprofessional collaborative education [16]. IPE shows promise in improving patient outcomes and care delivery [17], but IPE research often fails to report learner-based outcomes, such as observable clinical skills [18]. With the exception of obstetrics [19–21], there is relatively little research examining IPE in teaching ultrasound. Combining near-peer or peer-led teaching and IPE strategies has been explored in anatomical education, mostly involving first-year medical students and physical therapy students [22–24]. To our knowledge, there has not been research exploring the use of peer-led IPE in teaching POCUS.

The goal of this study was to utilize diagnostic medical sonography (DMS) students as near-peer teachers in training internal medicine residents (IMR) to perform abdominal POCUS. DMS students have expertise in abdominal sonography and may represent a viable alternative to medical faculty in teaching POCUS to trainees. This strategy was evaluated quantitatively and qualitatively to assess learner outcomes, explore the participants’ experiences, and make improvements to future IPE sessions.

## Methods

Setting and Participants: The intervention took place at a Midwestern academic health system in April 2018. An interprofessional faculty team developed a 4-h workshop in which DMS students (*n* = 6) from the College of Allied Health Professions served as coaches for first-year IMR (*n* = 24) learning abdominal POCUS. DMS students complete a 12-month curriculum, including > 1100 h of clinical instruction, resulting in either a Bachelor of Science in Medical Imaging and Therapeutic Sciences degree or a post-Baccalaureate professional certificate in Diagnostic Medical Sonography. At the time of the IPE workshop, DMS students were in the final months of their educational program. IMR were in their first post-graduate year. They had participated in cardiopulmonary and procedural POCUS workshops, but had minimal exposure to abdominal sonography.

Train-the-trainer Workshop: Prior to the workshop, DMS students participated in a 2-h train-the-trainer session to prepare them for their coaching responsibilities. The workshop design was informed by IPE practice recommendations, including use of interprofessional leadership, development of clear objectives, use of experiential learning [25], and emphasizing relevant non-clinical skills (such as communication) [18]. The session started with a 30-min didactic covering:

- Review of course objectives
- Definition and overview of IPE
- Principles of POCUS: definitions, clinical application, and terminology
- Background and training experiences of IMR
- Principles of adult learning with POCUS-specific examples
- Providing feedback utilizing the “ask-tell-ask” technique [26]

After the didactic session, DMS coaches participated in 4 simulation-based cases highlighting common POCUS coaching scenarios. Simulated cases included 2 DMS students, 1 simulated patient, 1 simulated learner, and 1 POCUS educational expert. DMS coaches and facilitators received instructional guides outlining their responsibilities and objectives. DMS students alternated between acting as the role-play coach and providing peer-feedback.

IPE Workshop: The IPE workshop covered ultrasound exam preparation and image acquisition of kidneys, bladder, and gallbladder. Table 1 outlines the course content. The course utilized a flipped-classroom approach with pre-course instructional videos used to maximize scanning time. Instructional videos were curated by course directors from free open-access online sources. The video run-time was 30 min and viewed by both IMR and DMS students.

**Table 1:** Course content of interprofessional point-of-care ultrasound workshop

POCUS Exam	Content
Exam preparation	• Room set-up: machine placement, bed positioning, dimming lights
	• Machine set-up: patient information, probe selection, exam-type
	• Patient set-up: communication, positioning, maintaining modesty
Right kidney	• Pre-scanning: labeling, probe positioning, site survey, techniques to aid image acquisition
	• Scanning: image optimization (depth, gain, centering), fanning organ in long and short axis, saving video loops
	• Anatomy identification: liver, hepatorenal recess, renal cortex, medullary pyramids, renal sinus
Left kidney	• Pre-scanning: labeling, probe positioning, site survey, techniques to aid image acquisition
	• Scanning: image optimization, fanning organ in long and short axis, saving video loops
	• Anatomy identification: liver, splenorenal recess, renal cortex, medullary pyramids, renal sinus
Bladder	• Pre-scanning: labeling, probe positioning, site survey
	• Scanning: image optimization, fanning organ in long and short axis, freezing image, measuring bladder dimensions, saving images
	• Bladder volume calculation
Gallbladder	• Pre-scanning: labeling, probe positioning, site survey, techniques to aid image acquisition
	• Scanning: image optimization, fanning organ in long and short axis, saving video loops
	• Anatomy identification: liver, gallbladder body and neck, main lobar fissure, portal vein

The workshop started with a 10 min didactic in which the course directors reviewed principles of IPE, discussed the background of the DMS students, and reviewed the day’s workflow. IMR dyads then rotated between 4 scanning stations. Each station was facilitated by a different DMS student-coach with a standardized patient serving as a live-model. Standardized patients were scanned by a faculty member prior to the workshop to ensure normal anatomy. Thirty minutes was allotted for each of the following stations: ultrasound exam preparation and right kidney, left kidney and bladder, gallbladder, and free-scanning time. DMS students received coaching checklists to facilitate teaching of course learning objectives. Faculty were available for technical problems, but otherwise did not participate. Participants used Phillips SPARQ (Andover, MA) and SonoSite Edge II (Bothell, WA) ultrasound machines.

Assessment: A mixed-methods approach was used to evaluate educational outcomes. Each participant was assigned a unique identifier to allow data tracking while maintaining anonymity. IMR completed an objective structured clinical exam (OSCE) immediately after the workshop to evaluate their image acquisition skills. The OSCE included evaluation of exam preparation, scanning technique, image quality, and image interpretation. Scanning technique and image quality was scored using a 47-item instrument (Additional file 1), with each item scored on a 3-point scale (0 = not performed or uninterpretable, 1 = partially performed or sub-optimal, 2 = fully performed or near-optimal). Participants also completed a 13-question image interpretation quiz, resulting in a maximum OSCE score of 107 points. Scanning technique was scored by an in-room faculty evaluator. Two blinded faculty members jointly scored saved OSCE images for quality following the workshop. Each scoring dyad was comprised of one DMS faculty member (KM or KW) and one internal medicine faculty member (CS or TM).

IMR and DMS student coaches completed online course evaluations developed from previously published research [27]. Responses were reported on a 5-point Likert Scale (1 = strongly disagree, 5 = strongly agree). For the OSCE and course evaluation, researchers calculated descriptive statistics, including mean scores and standard deviations, using Microsoft Excel (2016).

Finally, IMR and DMS students participated in semi-structured focus group interviews to provide researchers with an in-depth exploration of their experiences and attitudes towards the intervention. Interviews were conducted using an interview guide developed by the research team (Additional file 2). Each focus group had 5–6 participants and lasted approximately 45 min. Using a framework of qualitative description, data was analyzed by two coders using qualitative content analysis. Qualitative description is a method that offers “a comprehensive summary of an event in the everyday terms of those events,” and is “the method of choice when straight descriptions of phenomena are desired.” [28, 29]. One coder analyzed the data working directly from the audio recordings. A second coder analyzed the data working from transcripts of the recordings generated by automated transcription software. The transcripts were reviewed for accuracy before coding. Inter-coder agreement was calculated as a method for establishing reliability between coders [30]. Data was categorized into themes based upon focus group interview topics. Results of the qualitative analysis were validated via member checking in which a subset of participants were asked to review the analysis and verify that the resulting themes accurately represented the discussion in their group [31]. This study was approved by the University of Nebraska Medical Center Institutional Review Board (#704–17-EX).

## Results

Twenty-four of 24 (100%) IM residents completed the OSCE, with results presented in Table 2. Residents’ average composite OSCE score was 97.7/107 points (91.3%) (SD 5.2). Organ-specific exam sub-group scores ranged from 84.9% (20.4/24) for the gallbladder to 94.2% (17/18) for the right kidney. IMR performed similarly on the scanning technique (57/62, 92%) and image quality (28.5/32, 89%) sub-sections.

**Table 2:** Objective simulated clinical exam scores for internal medicine residents following an interprofessional point-of-care ultrasound workshop

	Scanning Technique Sub-score			Image Quality Sub-score			Total Score		
	Mean Score (SD)	Max Score	%	Mean Score (SD)	Max Score	%	Mean Score (SD)	Max Score	%
Exam Prep	16.8 (1.4)	18.0	93.1	NA	NA	NA	16.8 (1.4)	18.0	93.1
Right Kidney	9.7 (.9)	10.0	97.0	7.3 (.8)	8.0	91.3	17.0 (1.1)	18.0	94.2
Left Kidney	9.4 (1.5)	10.0	94.0	7.0 (.8)	8.0	88.0	16.4 (1.8)	18.0	91.2
Bladder	7.8 (.4)	8.0	97.4	7.1 (.7)	8.0	89.1	14.9 (1)	16.0	93.2
Gallbladder	13.4 (2.7)	16.0	83.6	7.0 (1.2)	8.0	87.5	20.4 (3.4)	24.0	84.9
Image Quiz	NA	NA	NA	NA	NA	NA	12.3 (.9)	13.0	94.5
Total	57 (3.9)	62	92	28.5 (1.9)	32.0	89.0	97.7 (5.2)	107.0	91.3

*SD* standard deviation

All IMR (24/24, 100%) and DMS students (6/6, 100%) completed the course evaluation, with results displayed in Table 3. None of the DMS students had prior teaching experience. IMR and DMS student responses were positive for all questions, ranging from 4 to 4.8 on the 5-point Likert Scale. IMR reported the workshop improved their clinical (mean 4.7, SD 0.5) and POCUS skills (mean 4.8, SD 0.4). They also felt the DMS students provided helpful feedback (mean 4.8, SD 0.4) and inspired learning (mean 4.5, SD 0.6). DMS students reported the workshop led to improved communication skills (4.7, SD 0.5), enhanced teaching skills (4.7, SD 0.5), and strengthened prior knowledge (4.5, SD 0.5).

**Table 3:** Course evaluation responses for internal medicine residents and diagnostic medical sonography student coaches. Scores reported using 5-point Likert Scale, 1 = strongly disagree, 2 = somewhat disagree, 3 = neither disagree nor agree, 4 = somewhat agree, 5 = strongly agree.

Internal Medicine Residents (*n* = 24)	Mean (SD)
My instructor created a non-threatening learning environment.	4.8 (.4)
My instructor had sound understanding of course content.	4.8 (.4)
My instructor provided helpful feedback.	4.8 (.4)
My instructor inspired me to learn.	4.5 (.6)
My instructor was a role model for professionalism.	4.7 (.6)
My instructor provided a mentorship role.	4.4 (.7)
My instructor inspired me to want to teach in the future.	4.0 (1)
This activity improved my clinical skills.	4.7 (.5)
This activity enhanced my skills in performing point-of-care ultrasound.	4.8 (.4)
I will apply the skills I learned to my clinical practice.	4.3 (.7)
I would recommend this activity to other residents in my program.	4.5 (.6)
Diagnostic Medical Sonography Students (*n* = 6)	
This activity improved my ability to communicate with colleagues in my discipline.	4.7 (.5)
This activity improved my ability to communicate with colleagues in other disciplines.	4.7 (.5)
This activity helped me develop my teaching skills.	4.7 (.5)
As a result of this activity, I am more likely to be involved in teaching in the future.	4.0 (.6)
This activity improved my organizational skills.	4.0 (.9)
This activity improved my clinical skills.	4.2 (.4)
This activity allowed me to consolidate previous knowledge.	4.5 (.5)
I would recommend this activity to other students in my program.	4.5 (.5)

Twenty-three of 24 residents (96%) and 6/6 DMS students (100%) participated in focus group interviews. Inter-coder reliability was 88%. Themes and representative quotes are shown in Table 4. To aid with clarity, we organized themes into 3 descriptive groups: the learning environment, scanning techniques, and recommendations for future IPE workshops. Residents reported that DMS students created a friendly learning environment and that the IPE approach was complimentary to faculty-led workshops. Whereas DMS student coaches were more proficient in acquiring images, faculty had a better understanding of the clinical applications of POCUS. IM residents and DMS students noted the step-wise approach to scanning highlighted in the pre-course videos was sometimes at odds with the more exploratory technique employed by the DMS students. Both groups highlighted the need for mutual understanding of expectations and shared terminology. They also suggested allotting more time for coaches to demonstrate each exam prior to IMR scanning.

**Table 4:** Themes and representative quotes from focus group interviews. IMR = Internal Medicine residents

Themes	Representative Quotes
Learning Environment	
IMR felt the DMS students created a positive learning environment.	“It made it easier to just try and see what happens and you fail. You have students teaching you and they’re very willing to work with you. It’s not like having a faculty [instructor]... It’s someone else who you know is also learning and it makes it more comfortable, definitely.” IMR
IMR felt the DMS coaches offered a unique, yet complimentary, skill set compared to workshops led by faculty. DMS students were more proficient at acquiring images, while faculty were better at recognize clinical relevance.	“When she did it, it was actually really helpful to understand and watch how she positions her hand, watch how she holds the probe, watch her fine tuning the images just to get that perfect shot. It’s hard doing it for the first couple of times to understand exactly how hard to push and how to exactly manipulate the probe. Since they do it every day, it seemed better to ask.” IMR “Using the ultrasound as an extension of our physical exam. But listening to it from the faculty’s perspective knowing that this is going to applicable. We’re not just learning this to check it off. We’re actually going to need this and rely on it to make clinical decisions was helpful to have from a faculty standpoint that makes sense.” IMR
Both groups felt it was important to ensure there was a clear mutual understanding of expectations and terminology prior to the workshop.	“I feel that the expectations of what our job [as instructors] was done nicely with the sheets [we were given] and the grading rubric. [We knew] exactly wat they wanted us to point out to make sure that we were covering everything. That was very helpful.” DMS “I don’t know if they had enough direction to know what our expectations were.. .. They were all ‘here’s the paper’ that they’re [IMRs] are going to be tested on. So I’m sure they were thinking like ‘let’s go down this list of stuff that they need to know’ and let’s check off the boxes rather than use it as a hand-on experience.” IMR “[The terminology] is not universal. Where they use short access, we use transverse. Like I said, their lingo is a little bit different.” DMS
Scanning Technique	
IMR and DMS students felt there was sometimes conflicting scanning strategies. IMR tended to favor the step-wise approach demonstrated in their pre-course videos, whereas DMS students favored a more exploratory strategy.	“I think it was kind of hard sometimes. Like it was nice that they watch those videos before but then sometimes they wouldn’t stray away from the video. They’d go where the video told them to go and not listen to what I was trying to say, like we can try to go more inferior posterior here or whatever. They won’t be very receptive because they kept trying to revert back to that video.” DMS “I feel like before when we have the lectures, they give us more like a step-by-step, like put it this way and then you turn this direction and you do this. You know it was more step-by-step and they’re [DMS instructors] are kind of like put it on and we’ll kind of see what happens and then just kind of adjusted to this regimen. I think it is kind of nice to have both sides of things but you know it’s like if I went into a new patient, then I would have to have a framework on how to approach it.” IMR
Recommendations	
Participants suggested that future IPE workshops should allot more time for DMS students to demonstrate their scanning technique for the IMR.	“Maybe a little bit more time or letting us show them maybe a couple of minutes. This is how we do it. And then maybe that would kind of help them too if they see us moving around and not just staying in one spot, maybe that will help them.” DMS “We can have the sonographer go through every organ.. [to].. show us how they obtain all their views, on all their windows, and then we’ll rotate…That way we can see six different techniques on how to obtain the same view and then we can take which one we thought works best for us.” IMR

*DMS* diagnostic medical sonographer

## Discussion

This pilot study found that an interprofessional workshop utilizing DMS students as near-peer teachers was an effective strategy for teaching abdominal POCUS to IMR. The intervention was beneficial to both learners and coaches, with improvements in participants’ knowledge, skills, and attitudes spanning the IPEC Core Competencies for Interprofessional Collaborative Practice - interprofessional values, roles and responsibilities, communication, and teamwork [16]. Residents felt the DMS students were capable teachers, and they performed well on the OSCE. The DMS students reported their role as educators improved their communication, teaching, and clinical skills. These findings have important implications, as they demonstrate how interprofessional collaboration may allow broader adoption of POCUS in medical education, especially when faculty expertise is limited.

In the qualitative analysis, IMR reported the IPE workshop was complimentary to their prior experiences with faculty-led workshops. Although DMS coaches were more proficient in image acquisition, faculty educators had a better understanding of the clinical relevance of POCUS. This makes sense, given a sonographer’s responsibilities are primarily related to image acquisition, not interpretation and clinical decision making. This finding suggests that IPE needs to be integrated with profession- or discipline-specific education, as content relevance is a pillar of adult learning [32]. In our broader POCUS curriculum, the basic image acquisition skills taught in the IPE course were reinforced by faculty-led didactics and clinical applications in patient care settings. In the future, we plan to examine a longitudinal IPE curriculum in which IMR rotate with sonographers to sustain and enhance their image acquisition skills.

While the overall feedback from workshop participants was positive, there were several lessons learned. IMR felt some DMS coaches were less prepared than others, resulting in an over-reliance on the facilitator checklist. This may speak to differing levels of confidence among the DMS students, who were novice teachers. It is also possible there was apprehension related to perceived interprofessional hierarchy and biases [33, 34]. These findings supports the need for thoughtful preparation of near-peer coaches who may be in a teaching role for the first time. It may also be helpful to allow coaches and learners to interact prior to the teaching session. For example, allowing time for socialization or “ice-breaker” activities may encourage a more relaxed teaching/learning environment [24].

Participants reported differing scanning strategies were sometimes problematic. Whereas residents favored the step-wise approach outlined in the pre-course videos, sonographers often employed a less restrictive, investigative strategy. We believe this divergence is related to differing levels of competence in performing abdominal sonography, as detailed in the Dreyfus and Dreyfus model of skill development [35]. The IMR were novice learners, who tend to be rule-driven and reliant on analytical problem-solving. This contrasts to the more expert DMS students, whose extensive experience with abdominal sonography allowed them to apply a more intuitive, exploratory scanning strategy. In developing the course, we attempted to minimize the impact of teaching inexperience and the “competency gap” between DMS coaches and IMR in several ways. DMS student coaches reviewed the pre-course online modules assigned to the IMR, which outlined a standard approach to image acquisition. Furthermore, the train-the-trainer session was designed to allow the DMS coaches to practice teaching strategies targeted at novice learners in a simulation environment. Despite these efforts, there are opportunities for improvement. In future sessions, we plan to better highlight this difference during the train-the-trainer session, so that DMS students can adjust their expectations and teaching styles to better meet the residents at their level of development.

This study had several limitations. It was conducted at a single site with few participants. Additionally, the study did not have a control arm, such as a faculty-led workshop. Future research will need to confirm these findings with additional learner groups in other settings.

The costs associated with replicating this intervention will vary based on local resources. Having access to previously purchased machines, our primary expense was standardized patients ($20/hour). Three of the authors (CS, TM, KM) had salary support through program directorships. Although not all residency programs share a campus with a sonography program, there are nearly 500 accredited DMS and cardiovascular technology programs (the latter offering training in echocardiography and vascular sonography) in over 40 states [36], meaning interprofessional collaboration is a viable option at many institutions.

## Conclusions

An interprofessional educational approach utilizing near-peer teaching was an effective method for teaching IMR to perform abdominal POCUS. Both DMS student coaches and residents benefited from the experience. This approach may offer an option for teaching POCUS to medical trainees when available faculty expertise is limited.

## Additional files

Additional file 1: OSCE Scoring Form Abdominal Ultrasound Exam Assessment Form. Scoring form used for objective structured clinical exam of abdominal point-of-care ultrasound. (DOCX 27 kb)

Additional file 2: Interview Guide Interprofessional point-of-care ultrasound focus group interview guide. Description of data: Interview guide for semi-structured focus group interviews. (DOCX 15 kb)

## Abbreviations

DMS: Diagnostic medical sonography
IMR: Internal medicine residents
IPE: Interprofessional education
OSCE: Objective structured clinical exam
POCUS: point-of-care ultrasound
