# Supplementary material for: Building a bigger tent in point-of-care ultrasound education: a mixed-methods evaluation of interprofessional, near-peer teaching of internal medicine residents by sonography students
Source: BMC Med Educ. 2018 Dec 27;18:321. doi: 10.1186/s12909-018-1437-2 (PMC6307233; doi:10.1186/s12909-018-1437-2)
Supplement: Supplementary file 1 — OSCE Scoring Form Abdominal Ultrasound Exam Assessment Form. Scoring form used for objective structured clinical exam of abdominal point-of-care ultrasound. (DOCX 27 kb) [file 12909_2018_1437_MOESM1_ESM.docx]

**Abdominal Ultrasound Exam Assessment Form**

Learner ID:_______________________

(First initial, 2-digit day of the month you were born, and last 2 digits of your primary phone number)

Evaluator:_______________________

**Station 1: Set-up and right kidney**

Instructions: Closely observe the learner and score the items listed using the scoring system below. You should help ensure that video loops are saved of all required target organs, but otherwise do not coach the learner during their image acquisition. You may help if technical or logistical arise (please document under “notes”). You do not need to score the items listed under “image optimization,” as this will be completed when images are reviewed after the workshop. When the learner has completed the assessment, end the exam and prepare the room and machine for the next person.

Scoring: Not performed/uninterpretable = 0 Partially performed/sub-optimal = 1 Fully performed/near-optimal =2

Exam Set-up (18 points)

| Room Set-up |  |
| --- | --- |
| Machine positioned appropriately (proximity to patient, height adjusted, etc.) |  |
| Bed positioned appropriately (appropriate height, HOB flat, foot rest pulled out) |  |
| Lights dimmed |  |
| Machine Set-up |  |
| Selects curvilinear probe |  |
| Labels image (right kidney) |  |
| Selects appropriate machine preset (abdominal or FAST) |  |
| Patient Set-up |  |
| Patient starts in supine positions |  |
| Communication: Introduced themselves and their role, describes exam to be completed |  |
| Exposes only areas of patient’s body necessary for scan - uses towels/sheets/gown to cover other areas |  |

Exam: Right Kidney (18 points)

| Scanning the Patient | |
| --- | --- |
| Probe placed at right mid-axillary line at level of xiphoid |  |
| Probe marker facing patient’s head |  |
| When kidney identified, rotates as necessary to get true longitudinal view (usually counterclockwise) |  |
| Fans through entire organ in longitudinal view (**save loop – facilitator may help**) |  |
| Rotates into short-axis view and fans through entire organ (**save loop – facilitator may help**) |  |
| Image optimization: |  |
| Target organ centered |  |
| Gain optimized |  |
| Depth optimized |  |
| Overall diagnostic quality for assessing hydronephrosis |  |
| Facilitator to end exam and enter information for next learner | NA |

Notes:

**Abdominal Ultrasound Assessment Form**

Learner ID:_______________________

(First initial, 2-digit day of the month you were born, and last 2 digits of your primary phone number)

Evaluator:_______________________

**Station 2: Left kidney and bladder**

Instructions: Closely observe the learner and score the items listed using the scoring system below. You should help ensure that video loops are saved of all required target organs, but otherwise do not coach the learner during their image acquisition. You may help if technical or logistical arise (please document under “notes”). You do not need to score the items listed under “image optimization,” as this will be completed when images are reviewed after the workshop. When the learner has completed the assessment, end the exam and prepare the room and machine for the next person.

Scoring: Not performed/uninterpretable = 0 Partially performed/sub-optimal = 1 Fully performed/near-optimal =2

Exam: Left Kidney (18 points)

| Scanning the Patient | |
| --- | --- |
| Probe placed at left posterior-axillary line at level of xiphoid |  |
| Probe marker facing patient’s head |  |
| When kidney identified, rotates as necessary to get true longitudinal view (usually clockwise) |  |
| Fans through entire organ in longitudinal view (**save loop – facilitator may help**) |  |
| Rotates into short-axis view and fans through entire organ (**save loop – facilitator may help**) |  |
| Image optimization: |  |
| Target organ centered |  |
| Gain optimized |  |
| Depth optimized |  |
| Overall diagnostic quality for assessing hydronephrosis |  |

Exam: Bladder (16 points)

| Scanning the Patient | |
| --- | --- |
| Probe placed near superior edge of pubic symphysis in transverse view and tilts until bladder is in view |  |
| Freezes image and measures transverse dimension (**save image –facilitator may help**) |  |
| Rotates to longitudinal view and measures cephalad-caudal dimension (**save image –facilitator may help**) |  |
| Measures depth of bladder (anterior-posterior dimension) in either transverse or longitudinal view (**save image –facilitator may help**) |  |
| Image optimization: Based on last image saved by learner |  |
| Target organ centered |  |
| Gain optimized |  |
| Depth optimized |  |
| Overall diagnostic quality for assessing bladder volume |  |
| Facilitator to end exam and enter information for next learner | NA |

Notes:

**Abdominal Ultrasound Assessment Form**

Learner ID:_______________________

(First initial, 2-digit day of the month you were born, and last 2 digits of your primary phone number)

Evaluator:_______________________

**Station 3: Gallbladder**

Instructions: Closely observe the learner and score the items listed using the scoring system below. You should help ensure that video loops are saved of all required target organs, but otherwise do not coach the learner during their image acquisition. You may help if technical or logistical arise (please document under “notes”). You do not need to score the items listed under “image optimization,” as this will be completed when images are reviewed after the workshop. When the learner has completed the assessment, end the exam and prepare the room and machine for the next person.

Scoring: Not performed/uninterpretable = 0 Partially performed/sub-optimal = 1 Fully performed/near-optimal =2

Exam: Gallbladder (24 points)

| Scanning the Patient | |
| --- | --- |
| Probe placed at midline near xiphoid process or right costal margin |  |
| Probe marker facing patient’s head, rocked cephalad (I.e. "heel" probe under costal margin) |  |
| Slide along costal margin toward RUQ to identify gallbladder |  |
| Demonstrates or describes techniques to aid image acquisition e.g. deep inspiration, rolling to lateral decubitus position (facilitator may prompt the learner) |  |
| Rotate and tilt probe as necessary to get longitudinal view |  |
| Gallbladder, portal vein with main lobar fissure visualized (“exclamation point” sign) |  |
| Fans through entire organ in longitudinal view (**save image – facilitator may help**) |  |
| Rotates into short-axis view and fans through entire organ (**save loop – facilitator may help**) |  |
| Image optimization: Based on last image saved by learner |  |
| Target organ centered |  |
| Gain optimized |  |
| Depth optimized |  |
| Overall diagnostic quality for assessing cholelithiasis |  |
| Facilitator to end exam and enter information for next learner | NA |

Notes
