# Supplementary material for: Building a bigger tent in point-of-care ultrasound education: a mixed-methods evaluation of interprofessional, near-peer teaching of internal medicine residents by sonography students
Source: BMC Med Educ. 2018 Dec 27;18:321. doi: 10.1186/s12909-018-1437-2 (PMC6307233; doi:10.1186/s12909-018-1437-2)
Supplement: Supplementary file 2 — Interview Guide Interprofessional point-of-care ultrasound focus group interview guide. Description of data: Interview guide for semi-structured focus group interviews. (DOCX 15 kb) [file 12909_2018_1437_MOESM2_ESM.docx]

Interprofessional point-of-care ultrasound focus group interview guide

Internal Medicine Residents:

Introduction: During this interview, we will ask you about your experiences working with the medical sonography student-coaches during the abdominal ultrasound workshop. The term “POCUS” stands for “point-of-care ultrasound.”

1. How did your learning experiences in the POCUS workshop today compare with faculty-led POCUS workshops you’ve attended?
   - Probing questions: In what ways was this training more or less effective?
2. In what ways did your impressions of medical sonographers change after the workshop?
3. What other comments or suggestions about the training do you have?

Diagnostic Medical Sonography Student Coaches:

Introduction: During this interview, we will ask you about your experiences working with the internal medicine residents during the abdominal ultrasound workshop. The term “POCUS” stands for “point-of-care ultrasound.”

1. How did you feel about teaching internal medicine residents POCUS skills?
   - Probing questions: What aspects of teaching residents POCUS skills did you enjoy? What did you find challenging?
   - Probing questions: In what ways did you feel adequately prepared to be a coach? In what ways did you feel underprepared?
2. In what ways did your impressions of physicians change?
3. What have you learned about your teaching skills through participation in this workshop? Your communication skills?
4. What other comments or suggestions about the training do you have?
